# Overview of HSS Steel Grades Development and Study of Reheating Condition Effects on Austenite Grain Size Changes

**DOI:** 10.3390/ma14081988

**Published:** 2021-04-15

**Authors:** Tibor Kvackaj, Jana Bidulská, Róbert Bidulský

**Affiliations:** 1 Department of Plastic Deformation and Simulation Processes, Faculty of Materials, Metallurgy and Recycling, Institute of Materials and Quality Engineering, Technical University of Kosice, Vysokoskolska 4, 04200 Kosice, Slovakia; jana.bidulska@tuke.sk; 2 Asian Innovation Hub, P.O. Box c6, 04023 Kosice, Slovakia; robert.bidulsky@asihub.org

**Keywords:** high-strength steels (HSS), reheating conditions, austenite grain size, normal grain growth, abnormal grain growth

## Abstract

This review paper concerns the development of the chemical compositions and controlled processes of rolling and cooling steels to increase their mechanical properties and reduce weight and production costs. The paper analyzes the basic differences among high-strength steel (HSS), advanced high-strength steel (AHSS) and ultra-high-strength steel (UHSS) depending on differences in their final microstructural components, chemical composition, alloying elements and strengthening contributions to determine strength and mechanical properties. HSS is characterized by a final single-phase structure with reduced perlite content, while AHSS has a final structure of two-phase to multiphase. UHSS is characterized by a single-phase or multiphase structure. The yield strength of the steels have the following value intervals: HSS, 180–550 MPa; AHSS, 260–900 MPa; UHSS, 600–960 MPa. In addition to strength properties, the ductility of these steel grades is also an important parameter. AHSS steel has the best ductility, followed by HSS and UHSS. Within the HSS steel group, high-strength low-alloy (HSLA) steel represents a special subgroup characterized by the use of microalloying elements for special strength and plastic properties. An important parameter determining the strength properties of these steels is the grain-size diameter of the final structure, which depends on the processing conditions of the previous austenitic structure. The influence of reheating temperatures (T_Reh_) and the holding time at the reheating temperature (t_Reh_) of C–Mn–Nb–V HSLA steel was investigated in detail. Mathematical equations describing changes in the diameter of austenite grain size (d_γ_), depending on reheating temperature and holding time, were derived by the authors. The coordinates of the point where normal grain growth turned abnormal was determined. These coordinates for testing steel are the reheating conditions T_Reh_ = 1060 °C, t_Reh_ = 1800 s at the diameter of austenite grain size d_γ_ = 100 μm.

## 1. Introduction

The development of microalloying high-strength steel (HSS) to create high-strength, low-alloy steel (HSLA) began after World War II when market requirements were defined by the price of the steel plates and strips used in shipbuilding, oil-and-gas transportation and other industries. Metallurgical companies had to respond to the reduced ability of the market to purchase steel plates and strips by reducing prices through lower production costs. One way to reduce costs was to shorten the production cycle by leaving out the normalization annealing operation, which also required modifications to the chemical composition of steels. To compensate for the increase in mechanical properties, the aim was to produce lighter steel by reducing its thickness.

In the 50th years of the last century in Europe were performed the steel rolling experiments at a single-phase austenite region with the controlled-finished rolling temperatures that were lower than for conventional rolling. The last pass was carried out nearly above the Ar3 line [1]. This technology is often called thermomechanical treatment (TMT), sometimes as rolling at a controlled temperature [2], but both terms have been replaced by controlled rolling (CR). CR is characterized by the fact that the last passes are carried out at low-finishing rolling temperatures, which results in lower production costs by removing normalization annealing and increases mechanical properties and toughness through reducing the thickness of the rolled plates and strips. The advantage obtained by controlling the austenite structure is the formation of strongly deformed pancake grains from CR. These have to be maintained by rapidly cooling the deformed austenite immediately after the last rolling pass in order to provide a controlled phase transformation to the ferrite. Rapid cooling has to prevent the growth of ferrite grains and achieve a fine-grained final structure [3]. The cooling strategy that controls the passage through the phase transformation regions is termed controlled cooling (CC).

Serious research into the physical–metallurgical properties of steel began in the 60 years of the last century in the U.K. at the University of Sheffield and the British Iron and Steel Association (BISRA) [4]. The aim of the research was to improve strength properties, ductility, impact toughness and weldability of steels by controlling of the following parameters:

– physical-metallurgical parameters: diameter of austenite and ferrite grain size, recrystallization, precipitation and phase transformations
– processing parameters: reheating temperatures, rolling temperatures and plastic deformations in the spontaneous recrystallization region of austenite, rolling temperatures and plastic deformations in the non-recrystallization austenite region, including the finished rolling temperatures, cooling rate from the finished rolling temperatures and optimization of chemical composition through alloying elements [5].

Research related to the search for a suitable chemical composition was very important for the following reasons:

(i). It had to achieve good weldability by reducing the value of the carbon equivalent (i.e., reduction of carbon content). Manganese replaced carbon for interstitial strengthening [6] because it has a six-times lower influence on the carbon equivalent than carbon [7,8,9]. The result of the research was a chemical composition of C–Mn mild steel with the content of basic elements C ≈0.2 wt.%, Mn ≈1.5 wt.%. This type of steel grade was later referred to as St52 (S355) which was the basis for the development of microalloyed steels containing Nb, V, Ti,
(ii). It had to improve strength and ductility. The author [1] stated that the low-cost alloying element niobium (Nb) with a content of 0.005–0.03% wt.% was used for the first time in 1958 as a microalloying element and had an effect on the formation of Nb carbides and nitrides. Vanadium (V) with a content of 0.08–0.1% wt.% was used before the commercial application of Nb. Both elements are aimed at improving the strength and plastic properties resulting from the precipitating and grain-refining effects. The titanium (Ti) content of 0.1 wt.% has an important effect on grain-size refining [10,11].

The influence of Nb on structure formation, mechanical properties and weldability has been widely discussed such as by authors [12,13,14,15,16,17,18,19,20]. Studies showed that Nb has a strong influence on the formation of austenite and ferrite structures. The austenite structure is affected by Nb depending on temperature as follows:

– reheating temperature: The level has an effect on the volume of undissolved precipitates, which retard the grain growth of austenite via their pinning effect to the grain boundary motion;
– middle temperature austenite region: This retards structure recovery and the dynamic recrystallization of austenite,
– low-temperature austenite region: The formation of deformation-induced precipitates and precipitates correspond to the thermodynamic conditions. These precipitates are responsible for retarding the recrystallization processes and forming the pancake structure, which is characterized by a high level of ferrite nucleation that affects the resulting diameter of the ferrite grains, and transformation temperature: The effect of precipitation strengthening resulting from precipitation at the phase interface and precipitation itself in ferrite.

The maximal strengthening effect on the yield strength resulting from the grain-size refinement of ferrite was observed with a Nb content of 0.04 wt.% and was stronger as a precipitation strengthening effect [14,21,22]. If the Nb content is more than 0.04 wt.%, then the deformation ability of the weld seam decreases with an increase in the portion of the precipitates in the weld [17,23].

The authors [12,14,21,24,25,26,27] studied the influence of V on steel properties. V content of 0.1–0.2 wt.% is considered to be effective for increasing strength. A stronger strengthening effect on the yield strength was observed from the precipitation increment and a lower increment from the grain-size refinement. 

The authors [12,14,25,28,29,30,31] studied the influence of Ti on steel properties. The strengthening effect on the yield strength resulting from grain-size refinement is considered to be effective if the Ti content is 0.8 wt.%. The strengthening effect on the yield strength caused by precipitation is effective when the Titanium content is higher than 0.8 wt.%.

The effect of the microalloying elements can be summarized as follows:

- effect on grain size refinement: Nb > Ti > V,
- effect on precipitation strengthening: V > Nb > Ti,
- regarding steelmaking technology, it is necessary to provide deep desulphurization and deoxidation of the melt because the activity of microalloying elements (Ti, V, Nb) defined by standard free enthalpy (∆GT^0^) to sulfur and oxygen are strong. The following inequalities apply to liquid steel: TiS > VC > NbC > TiC > VN > NbO > NbN > TiN.

In connection to the optimization of chemical composition, the technologies of CR and CC (controlled cooling from finished rolling temperature) for heavy plates and wide strips were working out. CR–CC is a method that differs from conventional rolling by controlling the structure.

(a) high-temperature austenite:
  – control of austenite grain growth by reheating temperature and holding time at this temperature,
  – control of the thermo-deformation regime in order to refine the diameter of the austenitic grain size by repeating the cycle" plastic deformation–recrystallization". This technique called "Recrystallization Controlled Rolling" (RCR),
(b) low-temperature austenite:
  – control of thermo-deformation regime to form elongated deformed (pancake) austenitic grains with the possibility of continual deformation into the two-phase region of austenite–ferrite,
(c) phase transformation from austenite:
  – controlled cooling from finished rolling temperature, i.e., a strategy of controlled transition through the area of phase transformations to obtain the required final structural composition.

A special type of CR is ferritic rolling (FR). FR is starting as RCR of austenite. With temperature decreasing RCR process immediately passes into the rolling of ferrite, followed by the final CC process [32].

**Figure 1:**
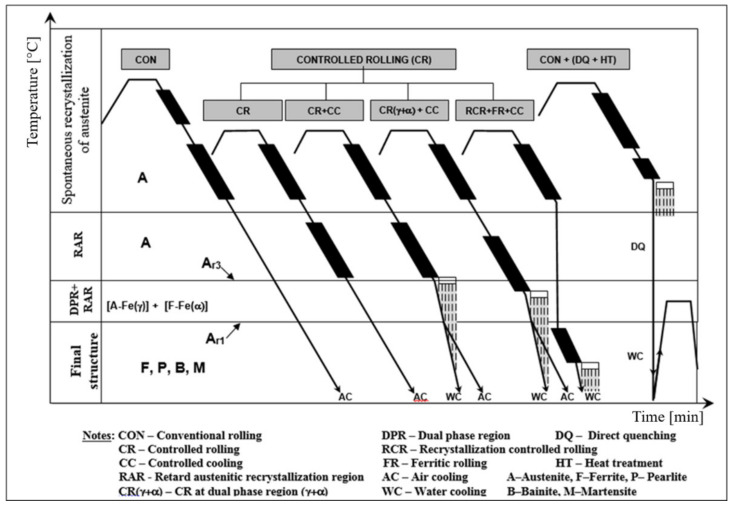
The scheme of temperature depending on plastic deformations processes.

CR–CC technologies can substitute heat treatment processes to some extent. The scheme of CR–CC processes, in comparison with conventional hot-plastic deformation and heat treatment, are shown in Figure 1.

The processes of the CR and CC of steel is still the subject of extensive study and analysis at scientific conferences and in journals and books [32,33,34,35,36,37,38,39,40,41,42,43,44,45,46,47,48,49,50,51,52,53,54,55,56,57]. Significant refining of the coarse grain (CG) structures of the polycrystalline materials can be achieved by the following procedures:

– thermoplastic deformation and cooling named as CR–CC (conventional methods of grain size refinement) with the diameter of the final grain size at the level of microns d ≥ 1 μm
– severe plastic deformation processes (SPD), which represent new methods of forming the final structure with grain-size diameter at the level of nanometers d < 1 μm [58,59,60,61].

**Figure 2:**
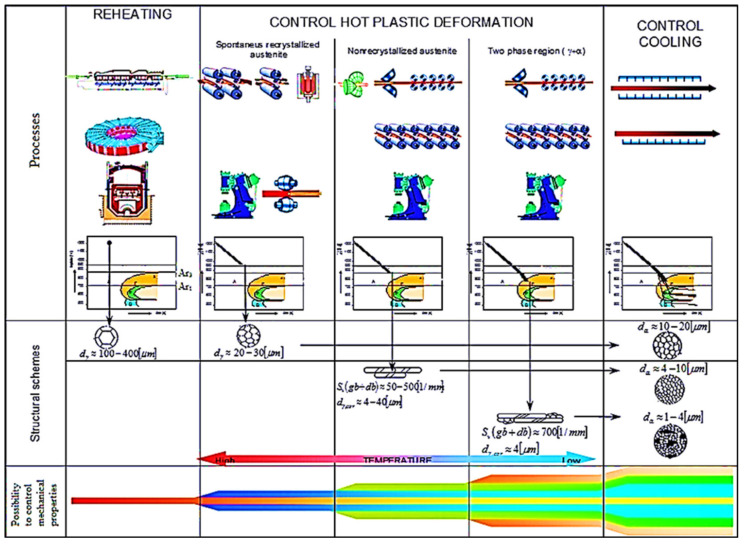
The scheme of structural formation by plastic deformations and phase transformations.

The influence of thermoplastic deformations carried out in the monophase region of austenite and the dual-phase region (γ+α), followed by the transformation of austenite on the final structures obtained by the RCR, CR and CC processes, is schematically given in Figure 2, which shows the following:

(i). direct phase transformation of austenite from reheating temperature produces very coarse-grained ferrite; the grain size depends on reheating condition.
(ii). plastic deformation conditions realized in
  – monophase austenite region at deformation temperatures.
  – spontaneous recrystallization of austenite—RCR [62,63,64] produced polycrystalline austenitic grains with diameter dγ ≈ 50–80 μm, which transformed to polycrystalline ferrite grain with diameter dα ≈10–30 μm,
  – non-recrystallization austenite region—CR [56,63] (narrowly raised Ar3 temperature) formed deformation-elongated austenitic grains with an effective ferritic nucleation surface calculated from grain boundaries and deformation bands Sv(gb+db) ≈ 25–500 1/mm [56]. This corresponds to the corrected diameter grains of austenite d_γ, cor_ ≈ 4–70 μm) transformed to polycrystalline ferrite with diameter d_α_ ≈ 2–10 μm.
(iii). dual-phase (γ+α) region—CR [55,63] (non-recrystallized austenite–deformed ferrite) bellow Ar3 temperatures with Sv(gb+db) ≈1000 1/mm, corresponding to d_γ, cor_ ≈ 2 μm with subsequent transformation to ferrite (non-recrystallized austenite, to polycrystalline ferrite–deformed ferrite, to ferrite subgrains) with diameter d_α_ ≈ 1–2 μm.

The described process methods of CR and CC for grain refinement are effective in the range of coarse-grain structures. Previous discussed methods leading to the refinement of structures below the limit value of <1 μm are ineffective. 

The steels that have excellent structural, toughness, fatigue, plastic and strength properties can be named progressive steels which are including HSS and AHSS [62,63,64,65,66,67,68].

**Figure 3:**
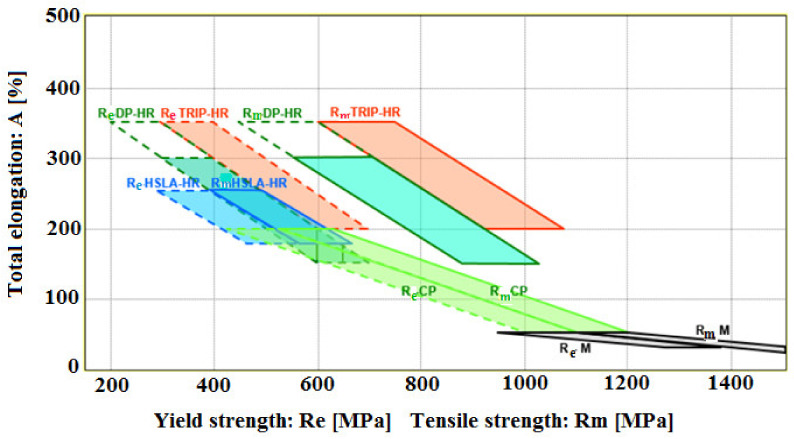
Scheme describing elongation depends on the strength properties for hot-rolled (HR) steel grades (HSLA, DP, TRIP, CP, M) (future development is indicated by chain lines).

The main difference between these steels is microstructure features. While HSS steels are characterized as single-phase ferritic steels with perlite reduced content, AHSS steels can be referred as more phase or multiphase steels containing more than just ferrite and perlite phases. AHSS steels may contain a mutual combination of two or more phases, formed by ferrite, bainite, martensite and residual austenite. The grain size refinement, solid solution strengthening and precipitation strengthening are acting as strengthening contributions for HSS. On the other hand, for AHSS these are the previous contributions supplemented by the strengthening contribution from the phase transformation effect. A special group of HSS are steels named high strength low alloyed steels (HSLA) with carbon content 0.04–0.25 wt.% and manganese 0.7–2.0 wt.%. The typical carbon content for steels applied to bending and welding is below 0.12 wt%. The base of these steels is grade St52 (S355) arranged on the chemical conception of C-Mn and yield strength up to 355 MPa. HSLA steels contained alloying elements such as chromium, molybdenum, nickel, copper, aluminum and first of all microalloying elements, as niobium, vanadium, titanium and zirconium [64,65]. According to authors [65], HSLA steel with a polycrystalline ferritic structure allows for reaching the yield strength close to 550 MPa, while the mixed ferritic-bainitic or bainitic structure shifts the yield strength to the level up to 700 MPa. Authors [62,66] stated that by combining controlled rolling carried out in the region of spontaneous recrystallization of austenite, which continuously runs to the two-phase phase region (γ–α), followed by rapid water cooling, it is possible to achieve a yield strength around 850MPa with a final two-phase ferrite–martensite structure. The AHSS group consists of the following steel grades: Dual Phase (DP) steel, Transformation Induced Plasticity (TRIP) steel, Complex (CP) steel, Martensitic (M) steel, Ferritic- Bainitic (FB) steel, Twinning Induced Plasticity (TWIP) steel [67,68,69,70,71,72]. The basic difference between HSLA and AHSS steel grades in terms of application is the source of ductility expressed by the ratio yield strength/tensile strength, as shown in Figure 3.

The dependencies show, that HSLA, CP and M steel grades are characterized by the narrow interrelationship of the yield strength and tensile strength parameters, while the DP and TRIP steel grades have these parameters largely separated. Therefore, HSLA, CP, M grades are more focused on strength and are suitable for structural components such as suspension systems, reinforcement, frames, longitudinal beams or chassis components. On the other hand, DP and TRIP steels have an excellent combination of strength and ductility and good energy-absorption capacity because of which they are suitable for use in structural parts and reinforcement [73,74,75,76,77,78].

**Figure 4:**
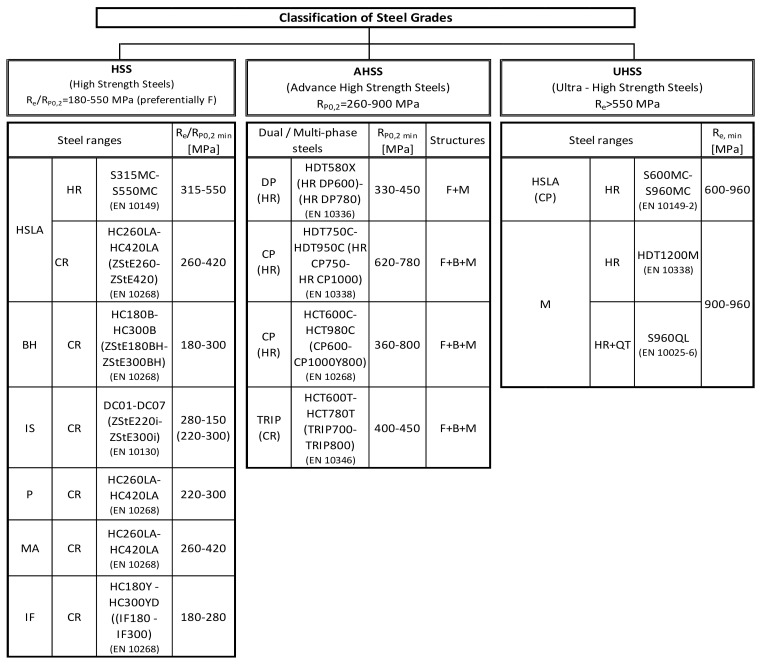
Classification of steel grades. Notes: BH—bake hardening, IS—isotropic, P—rephosphorized, MA—microalloyed, IF—interstitial, DP—dual phase, CP—complex phase, TRIP—transformation induction plasticity, M—martensitic, HR—hot rolled, CR—cold rolled, QT—quenched and tempered, F—ferrite, B—bainite, RA—residual austenite.

Characterization of steel grades according to yield strength for categories HSS, AHSS and UHSS are given in Figure 4. Within the group of HSS steels, HSLA steel represents a special subgroup characterized by microalloying elements such as Nb, V, Ti and Zr, which give it special strength and plastic properties.

As already mentioned, the base strengthening mechanisms of HSS and AHSS are grain refinement, solid solution strengthening, precipitation and transformation strengthening.

Grain refinement considered to be the most effective strengthening mechanism for the yield strength. The final diameter of the grain size strongly depends on the processing conditions of austenite, such as reheating, grain refinement during hot plastic deformation and phase transformations during material cooling.

*Grain-growth of austenite depends on reheating conditions.*

Austenite grain growth is a process by which either the mean grain size or second-phase particles increases. This process affects the change of mechanical and some other physical material properties. The grain growth of a coarse-grain (CG) polycrystalline materials is realized through the migration mechanism and coalescence of grain boundaries. The dominant driving force of grain growth is the decrease in grain boundary free energy. The increase in the average size of the grains is accompanied by a reduction in the total grain boundary energy per unit volume. Grain boundary migration cannot involve the nucleation of new grains but only the growth of existing ones. The grain growth of polycrystalline materials, then, depends on grain boundary migration. The driving force has tended to minimize grain boundaries and transform the initial polycrystalline state to monocrystalline. Opposing grain growth is the limited mobility of grain boundaries, which supports the development of a monocrystalline state [79,80]. The grain growth process can be divided into two types: normal grain growth and abnormal grain growth, also called secondary recrystallization. Microstructural changes during normal grain growth have a range of grain size and shape that are relatively uniform [79]. During abnormal grain growth, a few large grains grow by consuming smaller grains, causing a bimodal grain-size distribution.

Theories for normal grain growth in traditional CG polycrystalline materials the authors summarized as follows [81,82,83,84]:

– during grain growth in polycrystalline materials, there is a relatively narrow range of grain sizes and shapes;
– during grain growth, after sufficient time, the distribution of grain sizes becomes scaled to the mean grain size and remains self-similar;
– final grain-size distribution resulting from grain growth is generally insensitive to the initial distribution; and
– during grain growth, the mean grain size (radius) increases with time.

Taking into account the isothermal process, the following relation describes the kinetics of normal grain growth by means of a parabolic relationship given by Burke and Turnbull [85]:

(1) $$r^{n}-r_{0}^{n}=K\times t$$

(2) $$\mathrm{If}\;r^{n}\gg r_{0}^{n}\;\mathrm{so}\;r^{n}=K\times t$$

where: K = K_0_·exp(−Q_gg_/(R·T)); Q_gg_ is the activation energy of grain growth; R is the universal gas constant; T is the reheating temperature; K_0_ is a constant; n is the grain growth exponent (very often n = 2); r is the mean radius of grain size at time t; r_0_ is the mean radius of grain size in the initial state; and t is the time of grain growth.

In real metal materials the retarding effect of second-phase particles (e.g., oxides, carbides or nitrides) on the mobility of grain boundaries (low and high angle) is very often observed; therefore, second-phase particles have a strong pinning effect on boundaries. This effect in the literature is known as Zener drag. The influence of second-phase particles on grain growth has been studied by many authors [84,85,86,87,88]. Their pinning effect depends on volume fraction, size, interface, distribution and the thermal stability of particles. 

Microstructural changes during abnormal grain growth lead to the extensive growth of a few grains. Some grains can grow to several millimeters in diameter. The literature describes several factors influencing abnormal grain growth. The authors [89,90,91,92] describe abnormal grain growth is resulting from large pre-existing grains. Authors [93] assume that abnormal grain growth is achieved if the majority of grains are two to three times larger than the average size. Authors [94] studied the grain boundaries and triple junctions in a polycrystal and pointed out that the grain boundaries are high energy areas. The energy of a large number of atoms at the grain boundaries is large relative to the energy of atoms in the bulk of the grains. If the total excess energy of some part of the polycrystal relative to the energy of the grain boundaries and triple junctions exceeds the equilibrium level of energy, local abnormal grain growth can begin [95].

The mathematical description of austenitic grain growth over the entire range of reheating temperatures has been described by the authors [96] with a general equation of the form:

(3) $$d_{\gamma }^{n}-d_{\gamma ,0}^{n}=A\times t_{\mathrm{Reh}}\times \exp\left(\frac{-Q}{R.T_{\mathrm{Reh}}}\right)$$

where: n is the grain growth exponent (for steel n = 2.5); d_γ_ is the mean diameter of austenite grain size at time t_Reh_; d_γ,0_ is the mean diameter of austenite grain size in the initial state; t_Reh_ is the holding time on reheating temperature for grain growth; and T_Reh_ is the reheating temperature of grain growth.

The grain growth exponent for several metal materials was published by authors [97] as given in Table 1.

Another mathematical formula describing normal and abnormal grain growth may have the general form [98]:

(4) $$d_{\gamma }=a_{1}\times T_{\mathrm{Reh}}^{a2}\times t_{\mathrm{Reh}}^{a3}$$

where: a_1_, a_2_, a_3_ are regression coefficients depending on the reheating conditions; T_Reh_ (°C) is the isothermal reheating temperature; and t_Reh_ (s) is the holding time at T_Reh_.

The strengthening contribution resulting from the ferrite grain-size refinement can be primarily controlled very effectively by external processing factors, such as the reheating temperature and holding time at this temperature, which have a significant effect on changes to the diameter of austenite grain size. Controlling the diameter of the austenitic grains during reheating has an impact on the final diameter of ferrite grain size and will be discussed in the following sections.

## 2. Materials and Methods

Table 2 gives the local chemical analysis of several studied steel grades that are the result of long-term research by the author’s team together with major Slovak and foreign metallurgical companies that produce heavy plates, sheets, pipes and forgings for the automotive, machinery and energy industries [32,62,63,99]. Steel grade 38MnSiVS35 was used toproduce crankshafts for truck engines. The steel grade C–2.3Si characterized as a non-grain-oriented isotropic electric steel (NGOS) intended for the production of rotating electrical machines was investigated too. The electromotors produced from this steel grade can be used as driving units of electromobiles. The steel grades marked S380MC, S460MC and X70 are processed by CR–CC technology. While grades S380MC, S460MC are used as structural steel in various industries (e.g., machinery and automotive), X70 is tubular grade according to the API 5L standard.

Changes in the diameter of austenitic grains sizes depends on the reheating temperatures (T_Reh_), and the holding times at these temperatures (t_Reh_) were studied. The samples were reheated under the following experimental conditions: T_Reh_ ∈ <900;1250> (°C) with the step ∆T_Reh_ = 50 (°C) and t_Reh_ ∈ <600;3600> (s) with the step Δt_Reh_ = 600 (s). Light optical microscopy was used to evaluate the microstructures. The diameter of the austenite grain size was evaluated by the Jeffries planimetric round method. Mathematical analysis and numerical statistical methods were used to describe the experimental data in the form of equations.

## 3. Results and Discussion

### 3.1. Physical-Metallurgical Substance of Grain Growth Depending on Reheating Conditions

**Figure 5:**
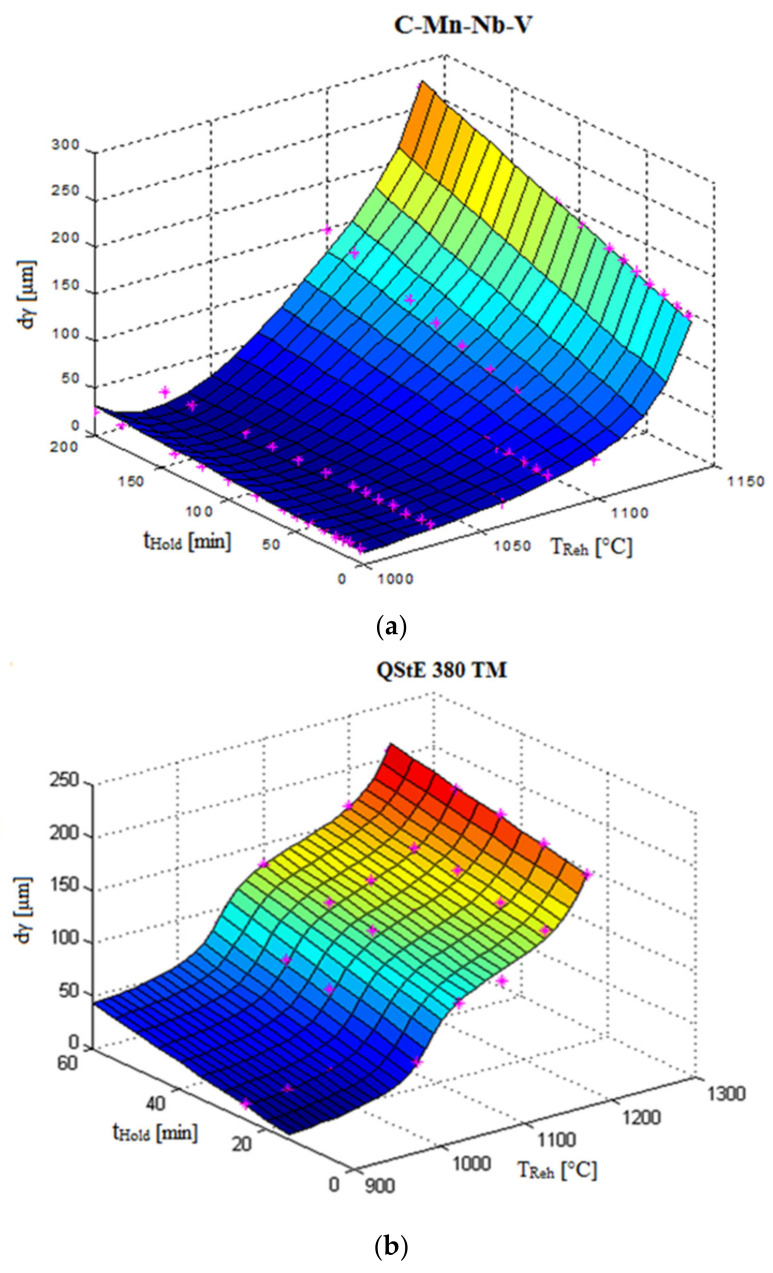

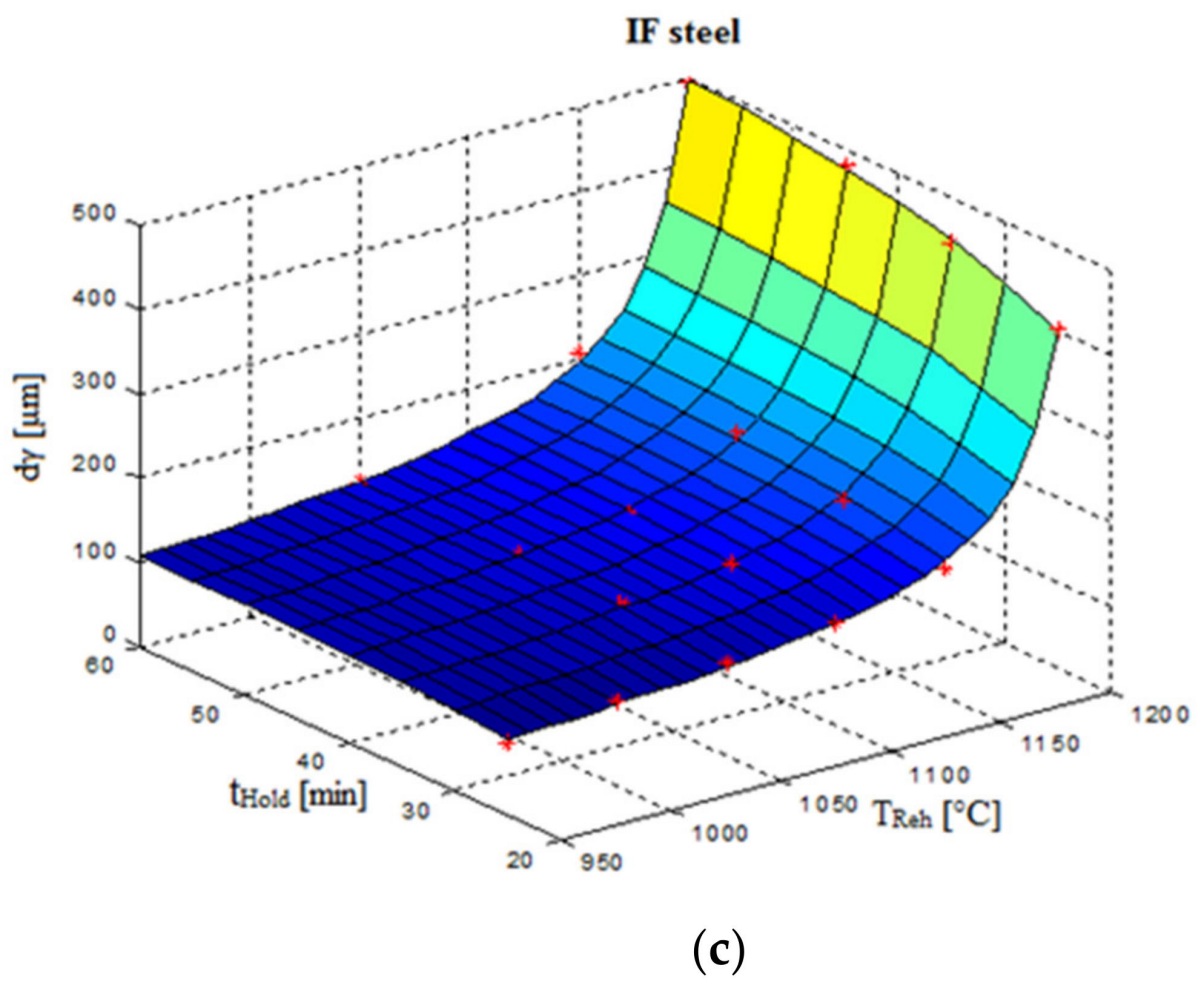
Graphical dependencies of austenite grain size diameter on temperature and time for several steel grades (**a**) C–Mn–Nb–V HSLA steel; (**b**) QStE 380 TM HSLA steel and (**c**) IF steel.

Reheating conditions (temperature and holding time at temperature) are important parameters for the structure formation before the plastic deformation processes. The influence of the reheating temperature and holding time on the changes of austenite grain size diameter for several types of steel grades was investigated. The results describing the dependence d_γ_ = f(T_Reh_; t_Reh_) are given in Figure 5.

**Figure 6:**
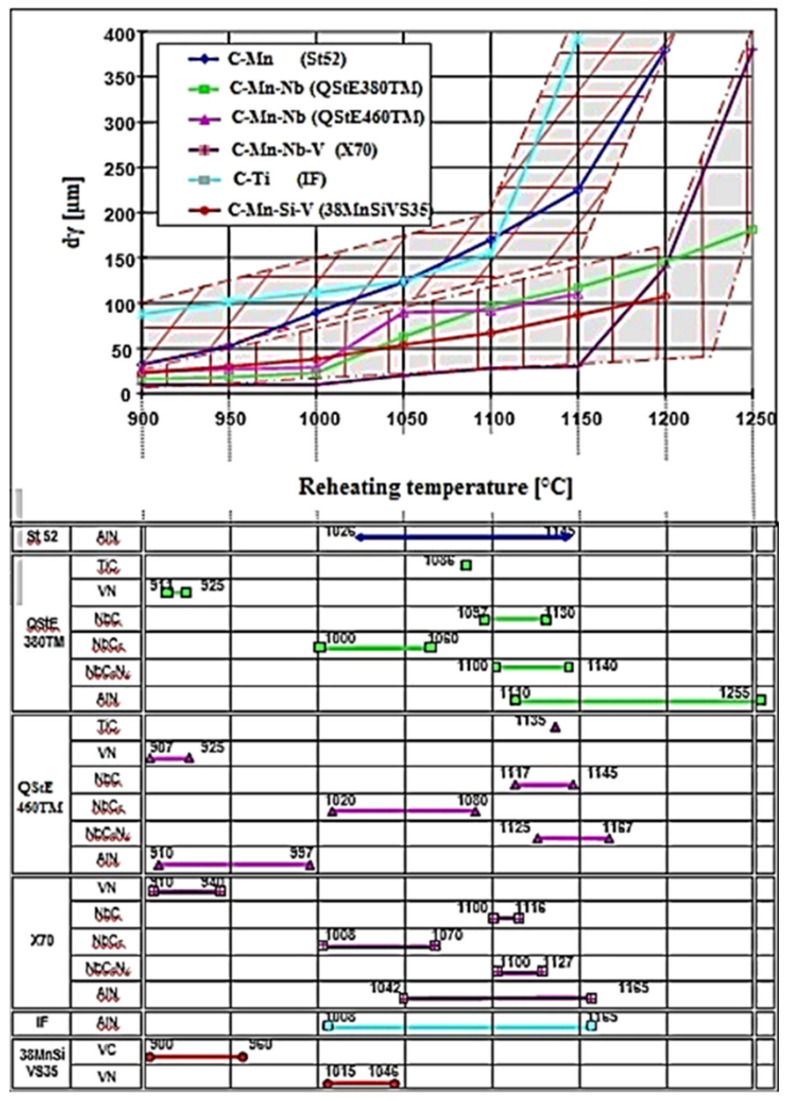
Austenite grain growth in depending on reheating conditions and solubility of precipitates.

Calculation of the dissolution temperature of precipitates as a function of thermodynamic constants and chemical composition is described by the following formula:

(5) $$T=B/\left(\log\left({\mathrm{Me}}_{x}{\left(C,N\right)}_{y}\right)-A\right)$$

where: Me (wt. %) is the precipitate metal component; C (wt.%) is the carbon content; N (wt. %) is the nitrogen content; A, B (-) are the thermodynamic constants; and T (°C) is the dissolution temperature of the precipitate. A detailed description of the solubility of precipitates is discussed in [98]. Graphical interpretation of the effect of reheating temperature and holding time on the solubility of precipitates and changes in austenite grain size diameters is shown in Figure 6.

**Figure 7:**
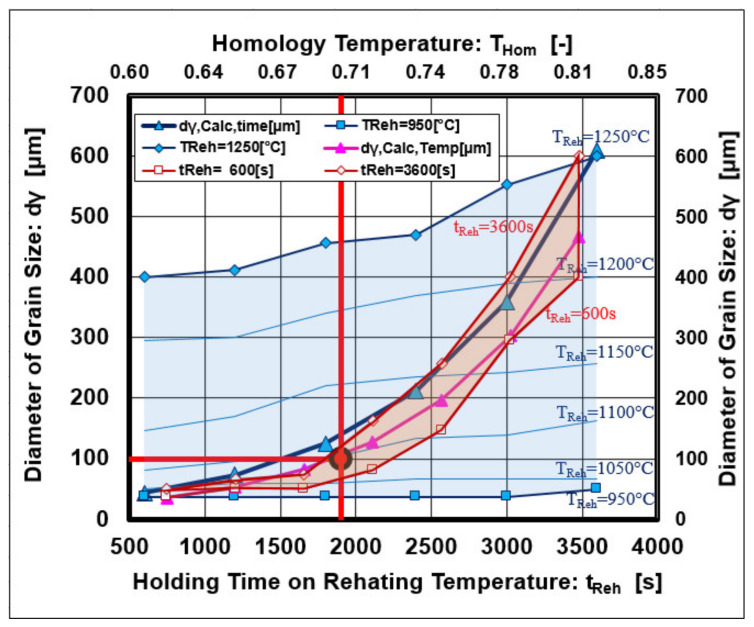
Isothermal austenite grain growth depends on reheating conditions.

Figure 6 shows that the strongest braking effect on austenitic grain growth was the result of Nb precipitates. The change in grain size is closely related to the coarsening or dissolution of the carbonitrides [98]. Steel grades without Nb showed higher values for austenite grain-size diameter, and the start of abnormal grain growth shifted to lower temperatures compared to those of Nb-bearing steels. Figure 7 shows a graphical, two-parametric dependence d_γ_ = f(T_Reh_; t_Reh_) for HSLA steel (C–Mn–Nb–V).

**Figure 8:**
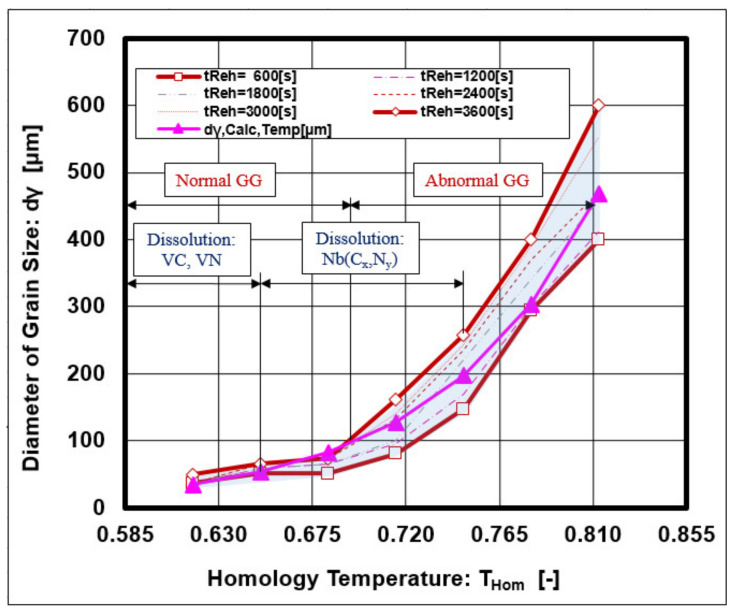
Isothermal austenite grain growth depends on the solubility of precipitates.

The graphical dependence given in Figure 8 shows that the VC and VN precipitates dissolved first at a temperature of 1000 °C, followed by the dissolution of complex precipitates Nb (Cx, Ny), which dissolved at 1150 °C. The reheating temperature showed a dominant effect on austenite grain growth, and a minor effect was observed from the holding time on temperature. 

From graphical dependences, the breaking point from normal to abnormal austenite grain growth was observed for a grain diameter d_γ_ = 100 μm, which lies in the coordinate axes T_Reh_ = 1060 °C; t_Reh_ = 1800 s.

**Figure 9:**
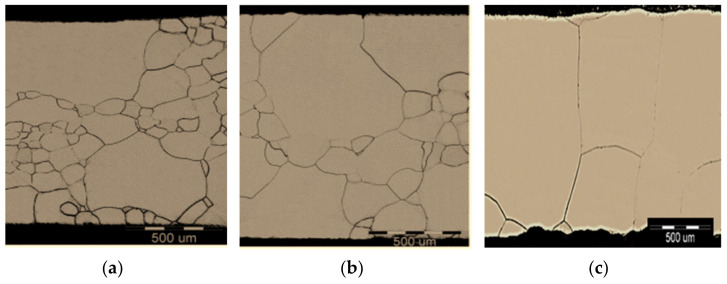
The stages of abnormal grain growth (steel grade: C–2.3Si) (**a**) T_Reh_ = 1050 °C, t_Reh_ = 1200 s, (**b**) T_Reh_ = 1100 °C; t_Reh_ = 1200 s, (**c**) T_Reh_ = 1100 °C; t_Reh_ = 2400 s.

The influence of reheating conditions on the development of microstructural changes was investigated by metallographic analysis. The different stages of abnormal grains growth with the possibility of the growth of several grains up to a millimeter are shown in Figure 9.

The study of grain growth confirmed that several grains can grow to abnormal dimensions depending on the reheating temperature and the holding time at that temperature. The rise in thermal conditions leads to an increase in the energy of the atoms at the grain boundaries and the triple junctions along with a subsequent dissipation of thermal energy into the local migration of the grain boundaries until an equilibrium energy state is reached and grain growth stops.

In the next part, the analysis focuses in detail on C–Mn–Nb–V steel as it represents a complex microalloyed grade with a wide spectrum of industrial uses.

### 3.2. Mathematical Description of Austenite Grain Growth

For C–Mn–Nb–V steel, general formula (3) [96] was modified to the following form:

(6) $$d_{\gamma }={\left[A+B\times t\times \exp\left(\frac{-Q}{R.T}\right)\right]}^{\frac{1}{n}}$$

where: A is the 14732.6 (μm), the constant including the initial diameter of grain size; B = 5.895.10^18^ (1/s), a constant; t (s) is the holding time on reheating temperature for grain growth; T (K) is the reheating temperature; d_γ_ (μm)is the mean diameter of austenite grain size at time t; n = 2.658 (-), a grain growth exponent; Q = 433 (kJ/mol), the activation energy; and R = 8.314.10^−3^ (kJ/(K.mol)), the universal gas constant.

Also, general formula (4) of the authors [98] that describes the development of the austenite grain size being dependent on the reheating condition for HSLA C–Mn–Nb–V steel has the real form:

(7) $$d_{\gamma }=2.068.10^{-36}\cdot T^{11.82}\cdot t^{0.2246}$$

Another formula describing the development of the austenite grain size depends on the reheating condition for HSLA C–Mn–Nb–V steel was derived to the form:

(8) $$d_{\gamma }=1.235\cdot 10^{7}\cdot \exp\left(\frac{-Q}{R.T}\right)\cdot \exp\left(\frac{t}{k}\right)$$

where k = 7452 (s) is a constant.

**Figure 10:**
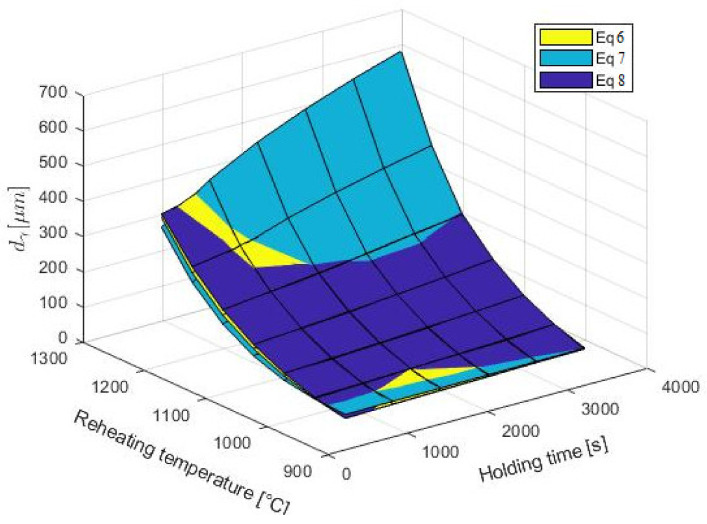
Geometrical interpretation of Equations (6)–(8).

A graphical interpretation of Equations (6)–(8) is shown in Figure 10.

The best correlation with the measured data was described by Equation (6), but Equations (7) and (8) also show a suitable correlation.

Equation (3) can be modified to the following form:

(9) $$\ln\left(d_{\gamma }^{n}-d_{\gamma ,0}^{n}\right)=\ln\left(A\times t_{\mathrm{Reh}}\right)-\frac{Q}{R}\times \frac{1}{T_{\mathrm{Reh}}}$$

or:

(10) $$\ln\left(d_{\gamma }^{n}-d_{\gamma ,0}^{n}\right)=\ln A+{\ln\;t}_{\mathrm{Reh}}-\frac{Q}{R.T_{\mathrm{Reh}}}=\left(\mathrm{lnA}-\frac{Q}{R.T_{\mathrm{Reh}}}\right)+\ln\left(t_{\mathrm{Reh}}\right)$$

**Figure 11:**
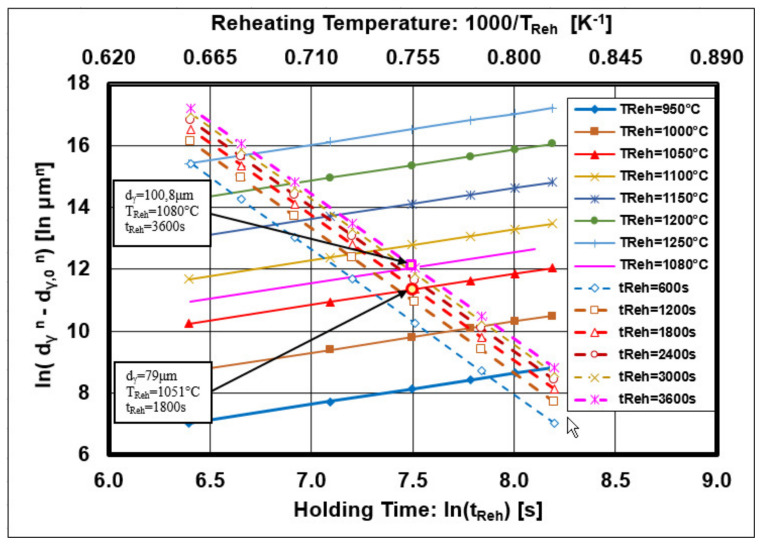
Graphical interpretation of Equations (9) and (10).

Equation (9) describes the function dependence ln (d_γ_^n^ − d_γ,0_^n^ = f(1/T_Reh_) and Equation (10) the function dependence ln (d_γ_^n^ − d_γ,0_^n^) = f(ln(t_Reh_)). These equations have a linear form with the graphical interpretation shown in Figure 11.

The intersection of the line describing the dependence ln (d_γ_^n^ − d_γ,0_^n^) = f(ln(t_Reh_)) for the constant temperature T_Reh_ = 1050 °C with the line ln (d_γ_^n^ − d_γ,0_^n^) = f(1/T_Reh_) for the constant t_Reh_ = 1800 s is at a point corresponding to the diameter of grain size ln (d_γ_^n^ − d_γ,0_^n^) = 11.5 μm^2.6^ (i.e., d_γ_ = 79 μm).

The intersection of the line describing the dependence ln (d_γ_^n^ − d_γ,0_^n^) = f(ln(t_Reh_)) for the constant temperature T_Reh_ = 1080 °C with the line ln (d_γ_^n^ − d_γ,0_^n^) = f(1/T_Reh_)) for the constant t_Reh_ = 3600 s is at a point corresponding to the diameter of grain size ln (d_γ_^n^ − d_γ,0_^n^) = 12.2 μm^2.6^ (i.e., d_γ_ = 100.8 μm). The analysis shows that the first slight increase in grain size diameter was in the interval d_γ_ ∈ <79;100> (μm) under conditions T_Reh_ ∈ <1050;1080> (°C) and t_Reh_ ∈ <1800;3600> (s). This first grain growth can be termed the pre-growth before the abnormal grain growth.

### 3.3. Overview of the Present Results

An overview of the development of chemical compositions and controlled process of rolling and cooling of steels to increase the mechanical properties and reduce the weight of hot rolled heavy plates and strips was presented. The steel-making process must respect that microalloying elements such as Nb, V and Ti have a strong affinity to oxygen and sulfur and therefore the melt must be desulfurized and deoxidized before alloying. The strongest strengthening effect to the yield strength for grain-size refinement and precipitation comes from Nb followed by Ti and V. Hot-rolling processes with control of thermo-deformation modes is called controlled rolling, and passage temperature controls through the phase transformations regions is termed as controlled cooling. 

The yield-strength values of the discussed steels lay in the following intervals: YS_HSS_ = 180–550 MPa; YS_AHSS_ = 260–900 MPa; and YS_UHSS_ = 600–960 MPa. In addition to the strength properties, the ductility of these steels grades is also an important parameter. AHSS-grade steels have the best ductility, followed by HSS and UHSS.

Future tasks for AHSS and UHSS grade steels will lead to applications where the dominant characteristic will be low weight and high strength not only for flat products but also for components with complex shapes and geometrical dimensions made by precision forging [100,101]. It will also be important to produce the details of complicated shapes, which cannot be made by precision forging, or by chip machining. The way to eliminate these discrepancies is powder metallurgy [102,103,104], which allows for the production of complicated products having a high level of strength and acceptable plastic properties. These new materials have the potential in powder metallurgy components used in the automotive industry (e.g., gear sets, connecting rods, and bearing caps, which require high surface hardness as well as good core toughness) and in machinery and energy [99,105]. On the other hand, today’s automotive industry is focusing on alternative fuels like hydrogen, where high-strength steel working at low temperatures is required to produce pressure vessels [106,107].

Historically, iron was a material that the Hittites processed as far back as 1200 years BC [108]. The modern processing of steel production dates to 1856 when Henry Bessemer patented a method of mass producing steel from molten pig iron [109]. Even though steel has evolved over 165 years, variations in the physical-metallurgical treatment of steel, with an emphasis on modifications to chemical compositions and subsequent processing of the solid phase, still allows for the creation of new steel properties for a wide range of applications. We suppose that developments in steel properties will be strongly determined by customer requirements that lead to the manufacturing of tailor-made material designs. These requirements must be met by intense physical-metallurgical research and precise identification methods as well as by modern software that describes changes in the internal structure of the material at the atomic level: molecular dynamics simulations that mimic elementary atomistic path-dependent processes by solving the equations of motion of all particles. Furthermore, software at the meso-dimensional level must be able to define the steel’s final properties, not only its structural and mechanical character but also its exact geometrical dimensions and shapes [110]. Nowadays, structure refinement is an attractive way to increase the strength properties of steels using the laser-powder bed fusion process [111,112,113,114].

## 4. Conclusions

Mathematical equations describing how the diameter of austenite grain size depends on reheating conditions were derived.

The rise in thermal conditions led to an increase in the energy of the atoms at the grain boundaries and the triple junctions with the subsequent dissipation of thermal energy into the local migration of the grain boundaries until an equilibrium energy state was reached until grain growth stopped.

The reheating temperature played an important role in changes to the austenite grain size. The research of the steel grade C–Mn–Nb–V showed that the abnormal grain growth of austenite strongly depends on the reheating conditions, which determine the solubility of the precipitate’s microalloying elements VC, VN, Nb (C_x_N_y_), thus reducing their braking effect on grain boundary migration. The breakpoint at which normal grain growth passes to abnormal growth was observed for the following conditions: diameter of grain size dγ = 100 μm and reheating conditions T_Reh_ = 1060 °C and t_Reh_ = 1800 s.

## Figures and Tables

**Table 1:** Grain growth exponent for isothermal grain growth in high purity metals.

Metal	Al	Fe	Pb	Sn
Exponent “n”	4	2.5	2.4–2.5	2.0–2.3

**Table 2:** The chemical composition of the studied HSLA and HSS steel grades (wt.%)**.**

Steel Grades		C	Mn	Si	Al	P	S	Nb	V	Ti
HSLA	QStE380TM (S380MC)	0.080	0.800	0.030	0.040	0.011	0.008	0.020	0.050	0.020
	QStE460TM (S460MC)	0.090	1.120	0.020	0.050	0.013	0.009	0.040	0.030	0.070
	X70 (C–Mn–Nb–V)	0.090	1.600	0.200	0.040	0.013	0.007	0.040	0.060	0.008
	38MnSiVS35	0.340	1.200	0.350	-	0.034	0.035	-	0.120	0.050
HSS	St52 (S355)	0.200	1.500	0.500	-	0.020	0.020	-	-	-
	IF	0.030	0.170	0.020	0.040	0.010	0.007	-	-	0.070
	C–2.3Si	0.015	0.250	2.300	0.464	0.018	0.004	-	-	-

Notes: IF—Interstitial free steel (EN10268), C–2.3Si—NGOES, 38MnSiVS35 (EN 10267).

## Data Availability

Data sharing is not applicable to this article.
